# Spatial Distribution, Ecological Risk Assessment and Source Analysis of Heavy Metals Pollution in Urban Lake Sediments of Huaihe River Basin

**DOI:** 10.3390/ijerph192214653

**Published:** 2022-11-08

**Authors:** Dun Wu, Hai Liu, Jian Wu, Xia Gao

**Affiliations:** 1 Key Laboratory of Intelligent Underground Detection Technology, College of Civil Engineering, Anhui Jianzhu University, Hefei 230601, China; 2 Enterprise Technology Center, Anhui ChaoYue Environmental Protection Technology Co., Ltd., Chuzhou 239000, China; 3 Public Geological Survey Management Center in Anhui Province, Hefei 230091, China; 4 Exploration Research Institute, Anhui Provincial Bureau of Coal Geology, Hefei 230088, China; 5 School of Architecture & Urban Planning, Anhui Jianzhu University, Hefei 230601, China

**Keywords:** spatial distribution, pollution source, ecological risk, heavy metals, sediments

## Abstract

Heavy metals in freshwater lake sediments often exist in various chemical forms. However, the investigation and evaluation of heavy-metal elements in the sediments of the study area have not been reported, and there is a lack of objective understanding of the concentration level of heavy-metal elements. Therefore, this study is the first to report the concentrations, sources, and potential ecological risks of heavy metals in the sediments of Chengdong Lake and Chengxi Lake in Huoqiu County, Anhui Province, China. The spatial distribution, pollution characteristics, potential pollution sources, and ecological risks of heavy metals in the sediments of Chengxi Lake and Chengdong Lake of Huoqiu City in the middle section of Huaihe River in Anhui Province, China have not been reported. In this study, the sediment samples of the two Lakes were collected systematically, and the concentrations of heavy metals (As, Cd, Cr, Cu, Hg, Ni, Pb, and Zn) were determined. The potential sources of heavy-metal elements in sediments were quantitatively analyzed according to the principal component analysis–absolute principal component fraction–multiple linear regression (PCA–APCS–MLR) receptor model. Descriptive statistics data showed that the enrichment degree of heavy metals in Chengxi Lake was higher than that in Chengdong Lake. The geo-accumulation index (*I_geo_*) and pollution load index (PLI) indicated that there was moderate pollution for Cu, As, Hg, Ni, and Zn. The calculation results of the potential ecological risk index (*E_r_*) of the two lakes indicated that Cd (*E_r__,_*_max_ = 92.22, *n* = 60) and Hg (*E_r__,_*_max_ = 64.39, *n* = 60) showed a certain potential ecological risk in a small amount of sediment, while other heavy metals were classified as low risk. The mean sediment quality guideline quotient indicated that there was a moderate degree of potential adverse biological toxicity in lake sediments. Spatially, the seriously polluted contamination zones were the central position of Chengxi Lake and the northeast end of Chengdong Lake. The PCA–APCS–MLR receptor model revealed that Cr, Ni, Cu, and Zn were mainly from natural sources while Cd, As, Hg, and Pb elements were mainly from industrial sources and pesticide sources.

## 1. Introduction

Lakes, rivers, and wetlands are important components of the terrestrial hydrosphere and play an important role in carrying the load between the layers of the earth’s surface system, and the utility model has the function of regulating the climate, improving the environment, and breeding aquatic organisms [1,2]. In recent decades, with the rapid development of industry and agriculture and the intensification of human activities, harmful substances have been discharged into lakes, rivers, and wetlands through various channels, resulting in the rapid deterioration of the ecological environment and frequent pollution. Among them, heavy metals are one of the most important pollutants in lakes, rivers, wetlands, and other ecosystems [3,4]. Heavy metals have attracted much attention due to their enrichment, concealment, persistence, and toxicity [5,6,7]. Moreover, heavy metals can be transferred through the food chain and accumulated over time, and when reaching a certain concentration level, they will cause harm to the central nervous system and circulatory system of organisms and pose a potential threat to human health and the ecosystem [8]. Therefore, it is important to study the source and pollution history of heavy metals in sediments.

Sediments are considered the largest pool of heavy metals in the aquatic environment [7]. Available studies have found that approximately 99% of heavy metals in aquatic systems eventually attach to sediment [9], and the concentrations of heavy-metal elements in sediment were typically 4 to 5 times higher than those in overlying water [10,11]. Sediment is an important component of the aquatic system and is not only an important base material for the growth of benthic organisms and aquatic plants but also functions as storage for heavy metals and other pollutants. When environmental conditions (reduction potential, pH, human disturbance, etc.) change, heavy-metal elements may be released from the sediment into the overlying water, resulting in secondary pollution and thus affecting the environmental quality of the lake water [12,13]. The concentration of heavy metals in sediments is not an isolated factor but rather interacts with the surrounding environmental factors. The study of the relationship between heavy metals and various environmental factors has been helpful to evaluate the effects of heavy metals on the ecosystem and elucidate the pollution characteristics of the local environment. Heavy-metal concentrations in sediments can reflect the status of the whole ecosystem polluted by heavy metals [14], and the measurement and evaluation of heavy-metal concentrations can help to understand the whole aquatic ecosystem polluted by heavy metals.

**Figure 1:**
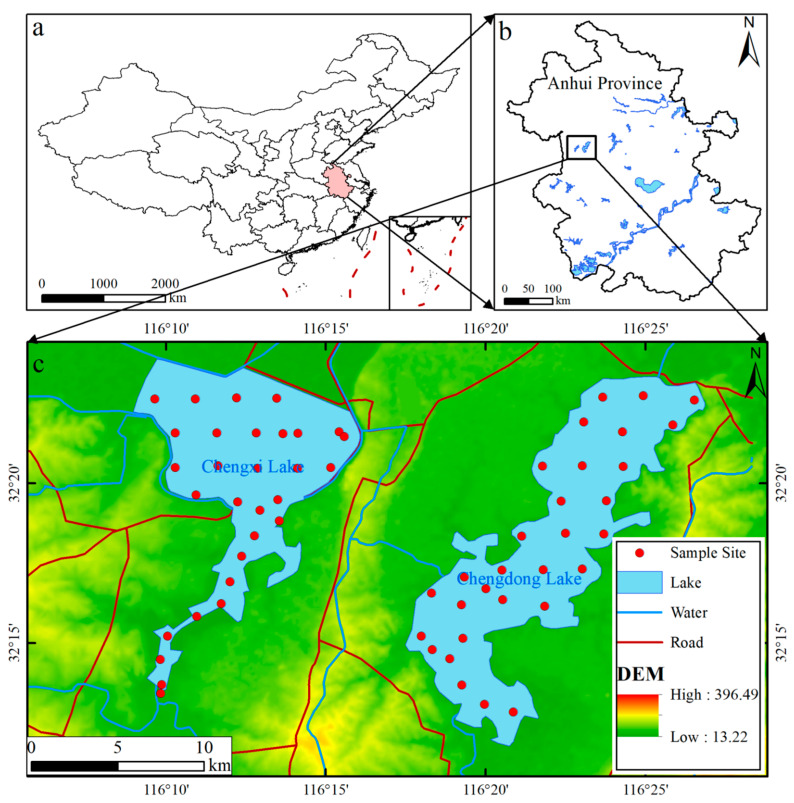
Location and distribution of sampling sites. (**a**) The geographical location of Anhui Province; (**b**) geographical location of the study area; (**c**) digital elevation models (DEM) around Chengdong Lake and Chengxi Lake and their relative positions. Spatial location of sampling points for lake sediment.

Huaihe River is located in the east of China, between the Yangtze River and the Yellow River, with a drainage area of 274,600 km^2^. The Chengdong Lake (3500 hm^2^) and the Chengxi Lake (10,700 hm^2^) of Huoqiu City, Anhui Province, are located on the south bank of the middle section of the Huaihe River. They are two large natural freshwater lakes in the Huaihe River system with an area second only to that of Hongze Lake. In recent years, with the rapid economic development of Huoqiu County, human activities (chemical fertilizer invasion, sewage discharge, industrial wastewater, urban construction, etc.) have intensified, causing varying degrees of pollution in the two lakes. Relevant studies demonstrate that because of the differences in different geographical environments and economic levels, the influencing factors of heavy-metal concentrations in lakes in different regions are not completely the same [6,7,9]. Freshwater lakes are one of the important resources for human production and life. Sediment is an indispensable and ecologically active part of riverbeds, in which heavy-metal elements exist in various chemical forms. There are no published data on the concentration of heavy metals in lake sediments in the study area (Figure 1), and no in-depth study has been carried out on the input of external pollution and the water quality of each monitoring section. Therefore, the purpose of this study is (1) to evaluate the concentration and distribution of heavy metals in the sediments of Chengdong Lake and Chengxi Lake of Huoqiu City; (2) to determine the pollution sources of heavy metals in sediments; and (3) to evaluate the pollution degree and potential ecological risk of heavy metals in sediment.

## 2. Materials and Methods

### 2.1. Overview of the Study Area

The study area is located in the northwest of Anhui Province at the northern foot of Dabie Mountain and on the south bank of the middle reaches of Huaihe River. The terrain of the study area is high in the south and low in the north, showing a trend of decreasing from southwest to northeast. The western part of the study area is hilly, the middle part is characterized by shallow hills and wavy plains alternating with hills and depressions, and the north and east are river lake plains. The highest peak in the area, Baida Mountain, is 419 m above sea level, and the lowest elevation in the low-lying area of Huaihe River alluvial plain is 18 m, with a relative height difference of 401 m. The annual average temperature in the area is 11–16 °C, the annual average precipitation is 600–1400 mm [15], the annual average evaporation is 1395.0 mm, the annual average sunshine hours are 2148 h, and the annual average frost-free period is 226 days.

The study area is located in the stratigraphic division of the southern margin of North China in the North China Stratigraphic Region. Most of the area is covered by the Quaternary system, and the exposed strata mainly include the Upper Proterozoic Sinian system, the Paleozoic Cambrian system, and the Jurassic and Cretaceous systems of Mesozoic. The main parent material is Cenozoic late Pleistocene sediment followed by a Cenozoic Holocene river, lake alluvial sediments, Yellow River alluvial sediments, and loess paleochannel alluvial sediments. The main soil types are fluvo-aquic soil and paddy soil. The land is comprised of mainly arable land, water areas, and water conservancy facilities, in addition to urban, industrial, and mining land.

### 2.2. Sample Collection and Testing

A grid layout was adopted and the basic sampling density was 1 point/4 km^2^, so as to collect Lake Bottom sediments. After the ship was stopped at the designated point by GPS navigation, a self-made sampler with a diameter of 5.5 cm was used for sampling. A continuous soil column of 0~40 cm was collected, and the sample weight was approximately 2.0 kg. A total of 60 sediment samples were collected from the lake bottom, including 30 samples from Chengdong Lake and 30 samples from Chengxi Lake (Figure 1).

The lake-bottom sediment samples were naturally dried in the shade, processed with a wooden hammer, and then passed through a 20-mesh sieve. The weight of the sample for inspection was approximately 200 g and the sub-sample was approximately 350 g, and which were then sent to the laboratory for testing.

The testing of 60 sediment samples was commissioned by the Anhui Geological Experimental Research Institute (Hefei Mineral Resources Supervision and Testing Center of the Ministry of Land and Resources). The concentrations of Cr, Ni, Cu, Pb, Zn, Cd, As, and Hg elements were measured according to China’s current standards. According to the DZ/T0279.1-2016 standard, the concentrations of Cr, Pb, and Zn in sediments were measured. The concentrations of Ni and Cu were determined according to DB34/T 2127.2-201. The concentrations of these five elements were measured using the X-ray fluorescence spectrometry (XFS) method. The determination of the Cd concentration was carried out according to the DZ/T0279.5.7-2016 standard, and the inductively coupled plasma mass spectrometry (ICP-MS) method was used. Both As and Hg concentrations were determined by atomic fluorescence spectroscopy (AFS) using the DZ/T0279.13-2016 and DZ/T0279.17-2016 standards, respectively. In addition, pH measurement was performed according to the ion-selective electrode method (ISE).

On the basis of the DZ/T0279.1-2016 standard, the detection limits of Cr, Pb, and Zn elements were 5 mg·kg, 2 mg/kg, and 4 mg·kg, respectively. The detection limit of Ni and Cu was 1 mg·kg according to the DB34/t 2127.2-201 standard. Based on the DZ/T0279.5.7-2016 standard, the detection limit of Cd was 0.021 mg·kg. The detection limit of As was 0.2 mg·kg according to the DZ/T0279.13-2016 standard. The detection limit for Hg was 0.005 mg·kg according to the DZ/T0279.17-2016 standard.

The instrumental parameters for the on-site determination of 8 elements’ concentrations are provided in the Supplementary Information.

### 2.3. Heavy-Metal Pollution Assessment Methods

#### 2.3.1. Geo-Accumulation Index ($I_{geo}$)

The geo-accumulation index ($I_{geo}$) was put forward by the German scholar Muller and can reflect the potential pollution degree of heavy metals in sediment or soil, and its calculation formula is as follows [16]:

(1) $$I_{geo}=\log_{2}\left(\frac{C_{n}}{KB_{n}}\right)$$

where $C_{n}$ is the concentration of a heavy-metal element in the sediment and $B_{n}$ is the background value of a heavy-metal element in the local sediment, of which the value is taken from the background value of the Yangtze River–Huaihe River basin of Anhui Province [17]. Background values of As, Cd, Cr, Cu, Hg, Ni, Pb, and Zn are 9.40, 0.104, 69.4, 24.9, 0.041, 25.0, 25.9, and 53.2 mg kg^−1^, respectively. K is the correction coefficient of the natural fluctuation of heavy-metal concentrations during diagenesis and is usually 1.5. Classification of the pollution degrees is presented in Table 1.

#### 2.3.2. Pollution Load Index (PLI)

The pollution load index (PLI) reflects the overall toxicity state of heavy metals [18] and its calculation formulas are as follows:

(2) $$\mathrm{PLI}=\sqrt[{n}]{CF_{1}\times CF_{2}\times CF_{3}\times \cdots \times CF_{n}}$$

(3) $$CF=\frac{C_{metal}}{C_{background}}$$

where n is the number of heavy metals to be analyzed; CF is the pollution index; $C_{metal}$ is the concentration of the corresponding heavy metals in sediment samples, in mg·kg^−1^; $C_{background}$ is the background value of heavy metals, in mg·kg^−1^. If PLI > 1, the soil is polluted by heavy metals [19]. The classification of pollution degrees is shown in Table 1.

### 2.4. Heavy-Metal Risk Assessment Methods

#### 2.4.1. Potential Ecological Risk Assessment ($E_{r}^{i}$)

The potential ecological risk index is used to evaluate the potential ecological risk level of heavy metals [20], and the calculation formula is as follows:

(4) $$RI=\overset{n}{\underset{i=1}{\sum }}E_{r}^{i}=\overset{n}{\underset{i=1}{\sum }}\left(T_{r}^{i}\times \frac{W_{i}}{B_{i}}\right)$$

where RI is the comprehensive ecological risk index; $E_{r}^{i}$ is the potential ecological risk index of heavy metal i; $T_{r}^{i}$ is the toxicity response coefficient of heavy metal i; $W_{i}$ is the measured value of heavy metal i, in mg·kg^−1^; and $B_{i}$ is the background value of heavy metal i, in mg·kg^−1^. Studies have shown that the toxicity response coefficients of the heavy metals Cu, As, Cd, Cr, Hg, Zn, Ni, and Pb are 5, 10, 30, 2, 5, 5, and 5, respectively [21]. Table 1 provides the classification standard of potential ecological risks of heavy metals.

#### 2.4.2. Mean Sediment Quality Guideline Quotient Method (*SQG* − *Q*)

The mean sediment quality guideline quotient (*SQG* − *Q*) focuses on the analysis of the toxic effects of heavy metals on aquatic organisms [22], and its calculation formulas are as follows:

(5) $$SQG-Q=\frac{\sum_{i=1}^{n}\left(PEL-Q\right)}{n}$$

(6) $${\left(PEL-Q\right)}_{i}=\frac{C_{i}}{PEL_{i}}$$

where PEL−Q is the possible effect concentration coefficient; $C_{i}$ is the measured concentration of heavy metal i, in mg·kg^−1^; and $PEL_{i}$ is the possible effective concentration of heavy metal i, in mg·kg^−1^. Generally, if SQG−Q≤0.1, it indicates that the area has not been polluted by heavy metals and has the lowest potential adverse biological toxicity; if 0.1<SQG−Q≤1, there is a moderate potential adverse biological toxic effect in the area; if SQG−Q>1, it indicates that there is a very high potential adverse biological toxic effect in the area [23].

### 2.5. PCA–ACPS–MLR Source Analysis Model

Principal component analysis (PCA), the absolute principal-component-score (APCS), and multiple-linear regression (MLR) were coupled to trace the heavy metals in lake sediments in the study area [24,25]. The main steps of PCA–APCS–MLR source analysis are as follows:

(1) Standardize the concentration data of heavy-metal elements and introduce a zero-concentration factor.

(7) Z0i=0−C¯iσi

(2) Calculate the PCA normalization factor fraction minus $Z_{0}$ to obtain the absolute principal component submultiple (APCS).

(3) Using APCS as an independent variable and the heavy-metal concentration as the dependent variable, multiple linear regression was carried out, and the source contribution of each heavy metal was calculated by the regression coefficient.

(8) $$C_{i}=b_{i0}+\sum_{p=1}^{p}\left(b_{pi}\times {\mathrm{APCS}}_{P}\right)$$

where ${\bar{C}}_{i}$ is the arithmetic mean of the heavy-metal concentration, in mg·kg^−1^, $\sigma_{i}$ is the standard deviation, $C_{i}$ is the estimated value of concentration, in mg·kg^−1^, $b_{i0}$ is the constant term obtained by multiple linear analyses of heavy-metal element i, $b_{pi}$ is the regression coefficient of source p to heavy-metal element i, and the mean value of $b_{pi}\times {\mathrm{APCS}}_{P}$ is the source p contribution value.

### 2.6. Experimental Data Processing

In this study, Excel 2021 was employed to carry out data processing and descriptive statistics of heavy metals in sediments, SPSS20.0 was used for correlation analysis and principal component analysis, and the related calculations of the PCA/APCS receptor model analysis were carried out in Excel 2021. The sampling distribution was completed using the ArcGIS10.8 platform. The pollution characteristic map of heavy-metal elements was obtained using the Kriging interpolation function of the geostatistical analysis module of ArcGIS10.8. The data relationship diagram was produced by Origin software.

## 3. Results and Discussion

### 3.1. Concentrations and Spatial Distribution Characteristics of Heavy Metals in Sediments

It can be seen from Table 2 that the average concentration of heavy metals in lake sediments in the study area was Zn > Cr > Ni > Cu > Pb > As > Cd > Hg from high to low, and the average concentrations (ranges) were 78.15 (48.1~104.6), 76.45 (56.7~97), 36.46 (23.7~46.8), 30.82 (21~39.6), 26.81 (20.2~36.4), 10.14 (5.43~18.56), 0.09 (0.04~0.32), and 0.03 (0.01~0.07) mg·kg^−1^, respectively. Table 2 shows that the average concentrations of Zn, Cr, Ni, Cu, Pb, As, Cd, and Hg in the sediments of Chengdong Lake and Chengxi Lake are 78.15 (*n* = 60), 76.45 (*n* = 60), 36.46 (*n* = 60), 30.82 (*n* = 60), 26.81 (*n* = 60), 10.14 (*n* = 60), 0.09 (*n* = 60), and 0.03 (*n* = 60) mg·kg^−1^, respectively. Considering that the mass fraction of each metal is different, it is inappropriate to indicate the enrichment degree or pollution level of elements in the order of their concentration. In this paper, the ratio of the average concentration of an element to its atomic weight was proposed as an enrichment factor to characterize the enrichment degree of an element. The results showed that the order of enrichment degree of the elements in Chengxi Lake was Cr (1.559, *n* = 60) > Zn (1.316, *n* = 60) > Ni (0.673, *n* = 60) > Cu (0.529, *n* = 60) > As (0.138, *n* = 60) > Pb (0.136, *n* = 60) > Cd (0.001, *n* = 60) > Hg (0.0001, *n* = 60); for Chengdong Lake, the order was Cr (1.382, *n* = 60) > Zn (1.074, *n* = 60) > Ni (0.569, *n* = 60) > Cu (0.441, *n* = 60) > As (0.132, *n* = 60) > Pb (0.123, *n* = 60) > Cd (0.0008, *n* = 60) > Hg (0.0001, *n* = 60). Statistics data showed that the enrichment degree of heavy metals in Chengxi Lake was higher than that in Chengdong Lake. The concentrations of heavy metals varied greatly, among which the coefficient of variation of Cd was 50.32%, with strong variability [26], indicating that the concentration of Cd was greatly affected by human activities; excluding Cd, the coefficient of variation of other elements was less than 50% (weak- to moderate-intensity variability), which indicates that the concentration of elements was less affected by human activities [26].

Compared with the concentrations of the upper continental crust (UCC) [27], the concentrations of all heavy metals in Chengxi Lake and Chengdong Lake were higher. In comparison to the average concentration of freshwater lake sediments in China [28], the concentrations of Ni, Cu, and Hg in the sediments of Chengxi Lake were higher while the average concentrations of all heavy metals in the sediments of Chengdong Lake were lower than the average concentrations of lake sediments in China. Compared with the average value of stream sediments in southern China and the average value of stream sediments in China [29], the concentrations of Cr, Ni, and Cu in the sediments of Chengxi Lake and Chengdong Lake were higher, while the concentrations of Cd, As, and Hg were lower. Excluding Cd and Hg, the concentrations of other heavy metals were higher than those of Chaohu Lake in Anhui Province [30], and excluding Zn, Pb, Cd, As, and Hg, the average concentration of other heavy metals were higher than that of Poyang Lake [30]; however, the concentrations of heavy metals in Chengxi Lake and Chengdong Lake were lower than those in Taihu Lake [31], Yangcheng Lake [32], Dongting Lake [28], and Tangxun Lake [33].

The concentrations of As and Ni in the sediments of Chengxi Lake and Chengdong Lake were higher than the TEL (Threshold Effect Level) value, the concentration of Ni in the sediments of Chengxi Lake was higher than the PEL (Probable Effect Level) value, the concentration of Cr in Chengxi Lake and Chengdong Lake were between PEL and TEL values, and the concentration of other elements were inferior to their corresponding TEL values. The above results indicated that As, Ni, and Cr elements in sediments had certain bio-toxicity effects [22], and more attention should be paid to the accumulation and toxicity of the above elements in aquatic organisms.

In a word, heavy-metal elements were enriched in the sediments of Chengxi Lake and Chengdong Lake. Restricted by many factors such as the geographical environment, soil parent material, and physicochemical properties of elements, the enrichment degree of different heavy-metal elements exhibited some differences. In addition, the degree of enrichment may be affected by human activities.

**Figure 2:**
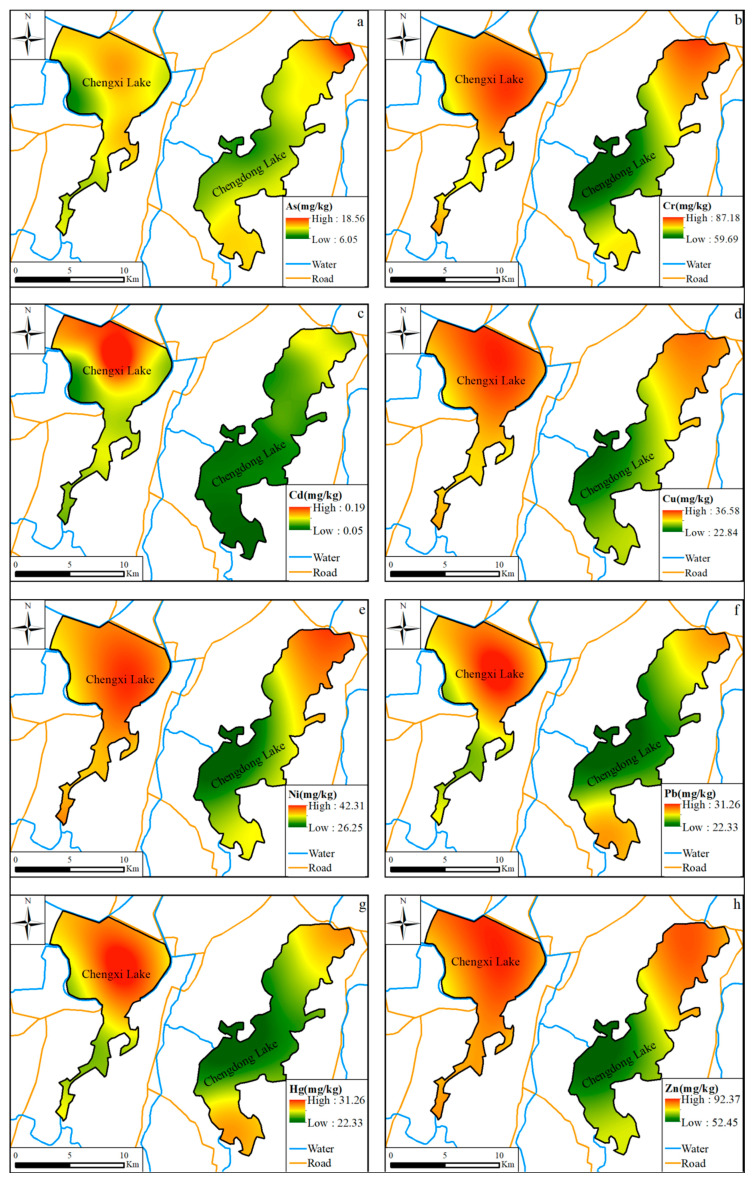
Spatial distribution of heavy metals in sediments of lake in study area. (**a**) As; (**b**) Cr; (**c**) Cd; (**d**) Cu; (**e**) Ni; (**f**) Pb; (**g**) Hg; (**h**) Zn. This figure was drawn with ARCGIS software.

The distribution of heavy metals in the sediment of Chengxi Lake and Chengdong Lake had obvious spatial heterogeneity (Figure 2). The spatial distribution of Cr, Cu, Ni, and Zn elements was consistent, and the high-value areas were located in the center of the Chengxi Lake and the northeast corner of the Chengdong Lake. As and Pb elements were relatively consistent in their spatial distribution, with the high-value area of Chengxi Lake mainly located northeast of the lake center while the high-value areas of Chengdong Lake were mainly located in the northeast and southwest corners. The spatial distribution of Cd and Hg concentrations showed relative uniqueness: The high-value areas of Cd were mainly located north of the lake center of Chengxi Lake and in the northeast corner of Chengdong Lake in a relatively uniform distribution, while the high-value areas of Hg were mainly located southeast of the lake center of Chengxi Lake and in the north of Chengdong Lake, showing a relatively uniform distribution.

### 3.2. Evaluation of Heavy-Metal Pollution in Sediments

#### 3.2.1. Geo-Accumulation Index ($I_{geo}$)

**Figure 3:**
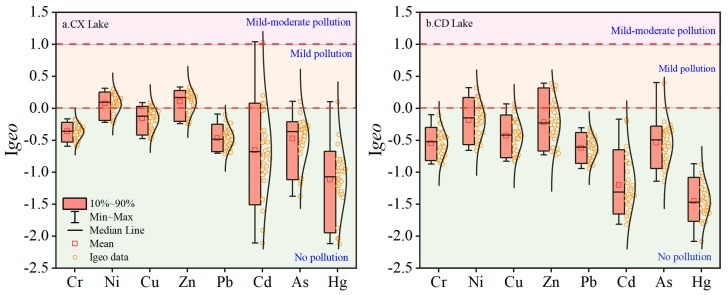
Boxplots of *I_geo_* of heavy metals in sediments. (**a**) Chengxi Lake; (**b**) Chengdong Lake.

The analysis results of the geo-accumulation index (*I_geo_*) are shown in Figure 3. The *I_geo_* of Cr and Pb in Chengxi Lake sediments and Cr, Pb, Cd, and Hg in Chengdong Lake sediments were all less than 0, which indicates that the degree of contamination can be considered uncontaminated. In the sediments of Chengxi Lake and Chengdong Lake, the *I_geo_* of Cu, As, and Hg in very few sampling points was greater than 0 and the *I_geo_* of Ni and Zn in most sampling points was greater than 0, which indicates that the pollution distribution of the heavy metals Cu, As, and Hg was limited, while Ni and Zn were the main pollution characteristic factors and were in a moderately polluted state.

In the sediments of Chengxi Lake and Chengdong Lake, 51.67%, 11.67%, 50%, 5.0%, 3.33%, and 1.67% of the sample points of Ni, Cu, Zn, Cd, As, and Hg were in a moderately polluted state. Ni and Zn pollution in the sediments of the two lakes was relatively serious, and the overall pollution level can be considered a moderate pollution state. The research area is an important iron ore production base in Anhui Province, China, and heavy-metal elements such as As, Pb, Cd, Cu, Zn, and Ni are the characteristic pollutants of metal mines [35]. Ni and Zn were the main heavy metals enriched in lake sediments, so one could speculate that the enrichment of heavy metals was greatly affected by mining activities.

#### 3.2.2. Pollution Load Index (PLI)

**Figure 4:**
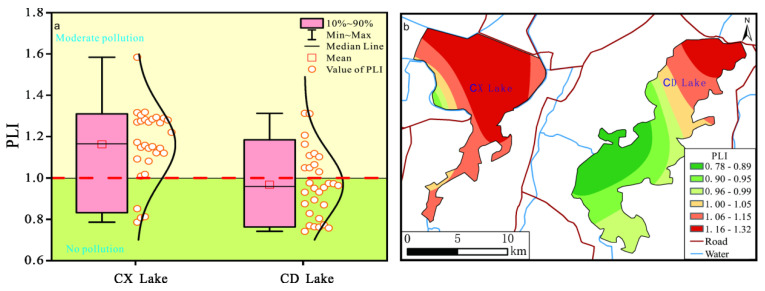
Boxplot (**a**) and spatial distribution (**b**) of PLI of heavy-metal elements in sediment. This figure was drawn with Origin 2017 software.

The average PLI value of sediments in Chengxi Lake and Chengdong Lake were 1.06 and 0.97, respectively. According to the classification of PLI (Table 1), 26 sample points in Chengxi lake were moderately polluted, with a pollution rate of 86.67%, and 11 sample points in Chengdong lake were moderately polluted, with a pollution rate of 36.67% (Figure 4a). As a whole, the pollution degree of Chengxi Lake was higher than that of Chengdong Lake.

According to the spatial distribution of PLI, most of the area in Chengxi Lake was moderately polluted and only the local area in the southwest corner was non-polluted. However, the polluted area of Chengdong Lake was mainly distributed in the northeast of the lake and the unpolluted area was mainly distributed in the southwest of the lake (Figure 4b).

### 3.3. Risk Assessments of Heavy Metals in Sediments

#### 3.3.1. Potential Ecological Risk ($E_{r}^{i}$*)*

**Figure 5:**
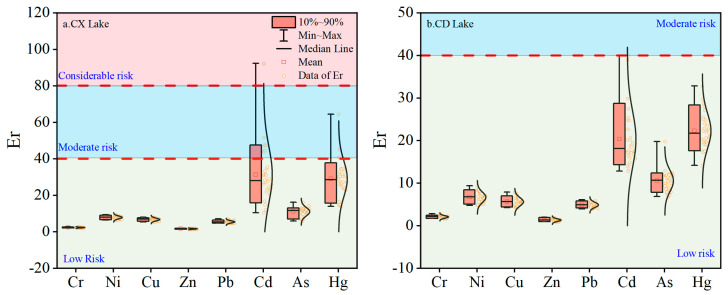
Boxplots of Er of sediment heavy-metal elements. (**a**) Chengxi Lake; (**b**) Chengdong Lake.

The potential ecological risk index ($E_{r}^{i}$) of lake sediments in Chengxi Lake and Chengdong Lake is shown in Figure 5. The potential ecological risk index of all heavy metals in Chengdong Lake sediments was less than 40 (Figure 5b), indicating that the ecological risk of Chengdong Lake was in a low-risk state. The ecological risk indexes of Cr, Ni, Cu, Zn, Pb, and As in the sediments of Chengxi Lake were all less than 40, of which five samples of Cd were at moderate risk, one sample was at considerable risk, and two samples of Hg were at moderate risk (Figure 5a).

The contribution of the potential ecological risk index ($E_{r}^{i}$) (Figure 5) mainly came from the elements Cd and Hg. Relevant research showed that Cd and Hg elements may originate from atmospheric transport, and the development of the social economy particularly increased the emissions of Hg, while Hg was easily affected by atmospheric transport, which led to an increase in input and sedimentation of Hg in sediments.

**Figure 6:**
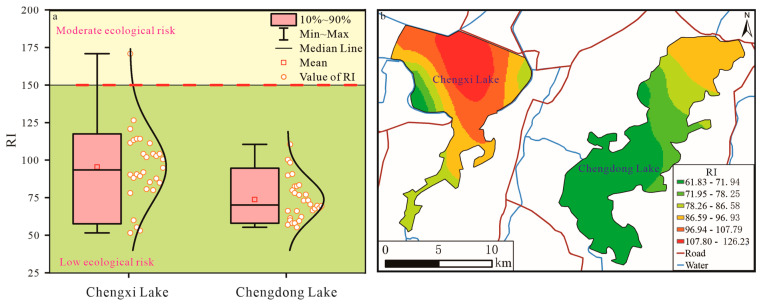
Boxplot (**a**) and spatial distribution (**b**) of RI of sediment heavy-metal elements.

The average values of the comprehensive ecological risk index (RI) of Chengxi Lake and Chengdong Lake were 95.43 and 73.76, respectively, showing a slight degree of hazard. It is worth noting that the RI value of one sample point in Chengxi Lake was 170.91 (Figure 6a), which was in a moderate degree of hazard, and this sample point was located in the center of Chengxi Lake. According to the spatial distribution of RI (Figure 6b), Chengxi Lake and Chengdong Lake showed a slight degree of hazard as a whole, but special attention should be paid to the center of Chengxi Lake, which may have a moderate degree of hazard or above (Figure 6b).

#### 3.3.2. Mean Sediment Quality Guideline Quotient (*SQG* − *Q*)

**Figure 7:**
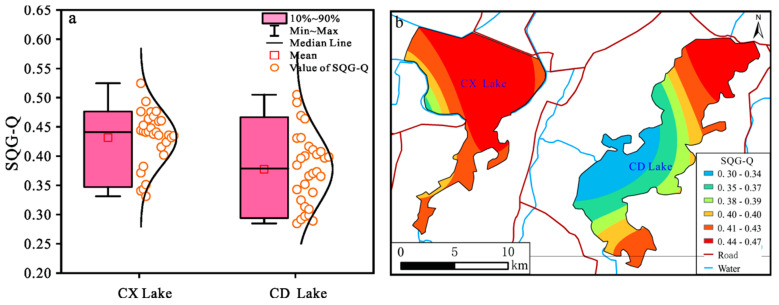
Boxplot (**a**) and spatial distribution (**b**) of *SQG* − *Q* of sediment heavy-metal elements.

The mean sediment quality guideline quotients (*SQG* − *Qs*) of Chengxi Lake and Chengdong Lake were 0.43 and 0.38, respectively (Figure 7a), which showed a moderate degree of potential adverse bio-toxicity. Cr, Ni, and As contributed greatly to the *SQG* − *Q* value, which may be influenced by the high background value. The overall *SQG* − *Q* value of Chengxi Lake was higher than that of Chengdong Lake, so we should focus on the potential adverse bio-toxicity of Chengxi Lake to prevent aquatic organisms from being affected by it.

### 3.4. Traceability of Heavy Metals in Sediments

#### 3.4.1. Correlation Analysis

**Figure 8:**
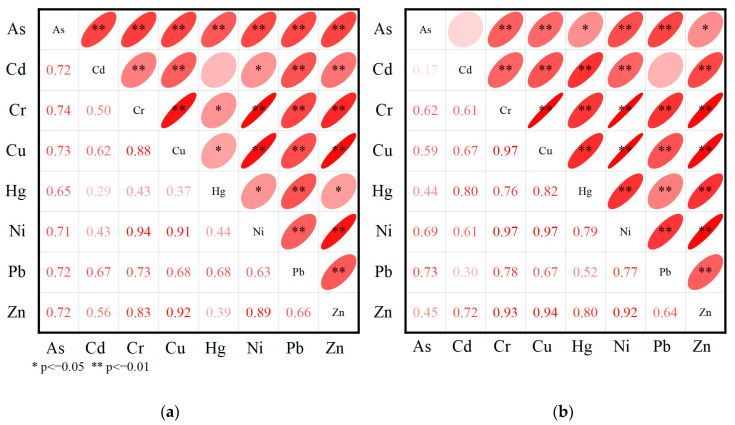
Plot of Pearson correlation coefficients. (**a**) Chengxi Lake; (**b**) Chengdong Lake.

The correlation of heavy metals in sediments is shown in Figure 8. There was a significant correlation (*p* < 0.01) between any two of the four elements Cr, Zn, Ni, and Cu in the sediments of the study area, and the correlation coefficient was as high as 0.97 (Cr-Ni-Cu), which indicated that the four elements Cr, Zn, Ni, and Cu had the same source. There was a strong correlation between any two of the four elements Pb, Cd, As, and Hg, indicating that Pb, Cd, As, and Hg may have the same sources [36].

#### 3.4.2. Principal Component Analysis

Eight heavy metals were examined by KMO and the Bartlett spherical test, and the KMO and Bartlett values were 0.850 and 910.34, respectively, with a significant level of 0, which indicated that the data were significant. Using Kaiser’s standardized orthogonal rotation method, two principal components could be extracted, accounting for 78.27% of the total variance (Table 3), which could explain most of the information on heavy metals in sediments.

The first principal component (PC1) explained 68.64% of the variance, mainly reflecting the component information of Cr, Ni, Cu, and Zn, and their pollution loads were 0.91, 0.92, 0.88, and 0.88, respectively. There was a very significant positive correlation between Cr-Zn-Ni-Cu demonstrating that their sources were similar. In addition, the coefficients of variation of heavy metals in the first principal component were relatively low and were less affected by human activities. Research showed that Cr, Zn, Ni, and Cu elements were mainly controlled by parent materials and soil-forming processes [36], and Cr elements in sediments were especially affected by soil-forming parent materials [37], indicating that the first principal component was mainly affected by soil-forming parent materials.

The *I_geo_*, PLI, and *E_r_* results of Cr showed that the sediments were not contaminated by Cr, which suggested that Cr in the sediments was less disturbed by external sources and was principally derived from the sediment parent material of the lake. Therefore, the first principal component represented a natural source.

The second principal component (PC2) explained 9.63% of the variance, reflecting the composition information of As, Cd, Hg, and Pb in sediments, and its pollution loads were 0.68, 0.81, 0.53, and 0.78, respectively. Relevant research has shown that elements such as As, Cd, and Pb mainly originated from human activities [31], and fertilizers and pesticides usually contain elements such as Cd, Pb, As, and Cd, which might show the impact of pesticides and chemical fertilizers and other pesticide activities [33]. Additionally, 45% of Hg in Chinese soil is from nonferrous metal smelting, 38% is from coal combustion, and 17% is from various other activities [26]. Coal combustion and nonferrous metal smelting were the main sources of atmospheric Hg pollution in China, accounting for >90% [35], and it entered the soil or sediment via atmospheric deposition [38]. The variation coefficients of Cd and Hg were the largest, which indicates that the spatial distribution of Cd and Hg was very uneven and obviously affected by human activities. Consequently, PC2 was mainly attributed to industrial and pesticide sources [39,40].

#### 3.4.3. APCS-MLR Source Analysis

The principal component analysis/absolute principal component fraction receptor model and the multiple linear regression equation (PCA/APCS–MLR) were used to quantify the contribution rates of two provenances to the heavy-metal concentrations in sediments, and this method had been proven to be very effective in research on heavy-metal source apportionment [41,42]. The calculation results of APCS-MLR are presented in Table 3. As can be seen from Table 3, the estimated values of eight heavy metals were all close to 1 compared with the measured values and had high *R* values, which indicated that the results of source analysis are remarkably reliable. In conjunction with the results of principal component analysis (source identification), it could be seen that the sources of heavy metals (Cr, Ni, Cu, and Zn) in lake sediments were mainly natural sources. The contribution rates of natural sources to the four heavy metals mentioned above were 57.09%, 59.46%, 55.31%, and 58.71%, respectively, followed by the contributions of industrial sources and agricultural sources regarding Cr, Ni, Cu, and Zn. Moreover, there were still some undetermined sources. Heavy metals (Cd, As, Hg, and Pb) in sediments mainly came from industrial sources and agricultural sources with contribution rates of 67.63%, 61.71%, 58.16%, and 76.20%, respectively, while the rest were natural sources and undetermined sources.

## 4. Conclusions

On the basis of this study, certain main conclusions were obtained. First, the enrichment degree of Zn, Cr, Ni, Cu, Pb, As, Cd, and Hg in Chengxi Lake was higher than that in Chengdong Lake. The concentration distribution of heavy metals in the two lakes had obvious spatial heterogeneity. The geo-accumulation index (*I_geo_*) indicated that Cu, As, Hg, Ni, and Zn in Chengxi Lake and Chengdong Lake were moderately polluted. However, the pollution load index (PLI) showed that only the scattered areas of Chengxi Lake were moderately polluted. The potential ecological risk index (*E_r_*) and integrated ecological risk index (RI) suggested that Chengdong Lake and Chengxi Lake were at slight ecological risk and had some potential biological toxic effects. The Cr, Ni, Cu, and Zn in Chengxi Lake and Chengdong Lake mainly originated from natural sources, the proportions of which were 57.09%, 59.46%, 55.31%, and 58.71%, while Cd, As, Hg, and Pb were mainly derived from industrial and agricultural sources with percentages of 67.63%, 61.71%, 58.16%, and 76.20%, respectively.

## Figures and Tables

**Table 1:** Description of geo-accumulation (*I_geo_*), pollution load index (PLI), and potential ecological risk index (*E_r_*).

***I_geo_***		**PLI**	
*I_geo_* ≤ 0	No pollution	PLI ≤ 1	No pollution
0 < *I_geo_* ≤ 1	No-moderate pollution	1 < PLI ≤ 2	Moderate pollution
1 < *I_geo_* ≤ 2	Moderate pollution	2 < PLI ≤ 3	Strong pollution
2 < *I_geo_* ≤ 3	Moderate-serious pollution	PLI > 3	Extremely strong pollution
3 < *I_geo_* ≤ 4	Serious pollution		
4 < *I_geo_* ≤ 5	Serious-extreme pollution		
*I_geo_* > 5	Extreme pollution		
*E_r_*		*RI*	
*E_r_* < 40	Low risk	RI < 150	Low ecological hazard
40 ≤ *E_r_* < 80	Moderate risk	150 ≤ RI < 300	Moderate ecological hazard
80 ≤ *E_r_* < 160	Considerable risk	300 ≤ RI < 600	Strong ecological hazard
160 ≤ *E_r_* < 320	High risk	RI ≥ 600	Extremely strong ecological hazard
*E_r_* ≥ 320	Serious risk		

**Table 2:** Descriptive statistics of heavy metals in the sediments of lakes in study area (mg·kg^−1^).

Parameters		As	Cd	Cr	Cu	Hg	Ni	Pb	Zn
Chengxi Lake (*n* = 30)	Minimum value	5.43	0.04	68.8	26.8	0.01	32.1	23.8	67.3
	Maximum value	15.18	0.32	92.5	39.6	0.07	46.5	36.4	100.4
	Average value	10.37	0.11	81.06	33.62	0.03	39.5	28.21	86.08
	Standard deviation	2.13	0.05	6.21	3.67	0.01	3.97	3.17	9.67
	Coefficients of variation (%)	20.57	49.22	7.66	10.91	33.61	10.05	11.23	11.24
Chengdong Lake (*n* = 30)	Minimum value	6.38	0.04	56.7	21	0.01	23.7	20.2	48.1
	Maximum value	18.56	0.14	97	39.1	0.03	46.8	31.4	104.6
	Average value	9.91	0.07	71.84	28.03	0.02	33.42	25.41	70.22
	Standard deviation	2.32	0.02	10.15	4.88	0.01	6.06	3.07	16.48
	Coefficients of variation (%)	23.38	32.65	14.13	17.4	18.48	18.12	12.09	23.47
Whole area (Chengxi Lake + Chengdong Lake) (*n* = 60)	Minimum value	5.43	0.04	56.7	21	0.01	23.7	20.2	48.1
	Maximum value	18.56	0.32	97	39.6	0.07	46.8	36.4	104.6
	Average value	10.14	0.09	76.45	30.82	0.03	36.46	26.81	78.15
	Standard deviation	2.22	0.05	9.55	5.12	0.01	5.93	3.4	15.6
	Coefficients of variation (%)	21.9	50.32	12.49	16.62	31.88	16.27	12.69	19.96
Chaohu Lake [30]		4.46	0.124	57.33	19.07	0.041	22.04	21.93	54.82
Taihu Lake [31]		13.34	0.479	102.46	44.71	0.109	45.5	37	163.62
Poyang Lake [30]		13.8	0.278	64.98	30.5	0.08	26.59	40.57	97.06
Yangcheng Lake [32]		15.85	0.45	101.28	66.54	0.09	68.72	34.02	187.33
Dongting Lake [28]		14.4	0.501	102	53.9	0.092	48.2	39	127
Tangxun Lake [33]		12.88	0.66	85.28	51.28	0.17	40.29	41.6	145.01
UCC [27]		1.5	0.098	35	25	-	20	20	71
Average value of lake sediments in China [28]		12.1	0.194	85	31.7	0.0053	36.8	31	88
Average value of stream sediments in southern China [29]		13.1	0.23	67	25	0.075	29	32.3	81
Average value of stream sediments in China [29]		12	0.18	61	23	0.046	26	27	71
TEL [34]		5.9	0.596	37.3	35.7	0.174	18	35	123
PEL [34]		17	3.53	90	197	0.486	36	91.3	315

Note: UCC—upper continental crust, TEL—critical effect concentration, PEL—possible effect concentration.

**Table 3:** Total variance of interpretation and rotating component matrix in the APCS–MLR model with metal sources in the lake sediments.

Elements	Initial Eigenvalue					Components		Rotating Component Matrices				
	Total		Variance Contribution Rates (%)	Cumulative Variance Contribution Rates (%)		PC1	PC2	PC1			PC2	
Cr	7.55		68.64	68.64		0.94	−0.30	0.91			0.39	
Ni	1.06		9.63	78.27		0.92	−0.33	0.92			0.35	
Cu	0.92		8.38	86.65		0.94	−0.26	0.88			0.42	
Zn	0.60		5.45	92.10		0.91	−0.30	0.88			0.37	
Pb	0.38		3.47	95.58		0.89	0.16	0.56			0.70	
Cd	0.19		1.70	97.28		0.74	0.43	0.28			0.81	
As	0.12		1.09	98.37		0.73	0.27	0.37			0.68	
Hg	0.10		0.87	99.24		0.62	0.16	0.36			0.53	
KMO and Bartlett’s Test												
Kaiser-Meyer-Olkin Measure of Sampling Adequacy				0.85								
Bartlett’s Test of Sphericity		Approx. Chi-Square		902.71								
		Degree of freedom		55								
		Significance		<0.001								
Elements		Contribution of sources		Undetermined sources	Measured values (O)		Forecasted values (E)		O/E	R^2^		Significance
		PC1	PC2									
Cr		0.5709	0.2440	0.1851	76.448		76.446		1.00	0.972		0.00
Ni		0.5946	0.2297	0.1757	36.457		36.458		1.00	0.966		0.00
Cu		0.5531	0.2605	0.1864	30.822		30.820		1.00	0.950		0.00
Zn		0.5871	0.2461	0.1668	78.150		78.154		1.00	0.913		0.00
Pb		0.3676	0.4594	0.1730	26.810		26.812		1.00	0.809		0.00
Cd		0.2302	0.6763	0.0935	0.089		0.090		1.00	0.739		0.00
As		0.3364	0.6171	0.0465	10.141		10.141		1.00	0.600		0.00
Hg		0.4006	0.5816	0.0179	0.026		0.026		1.01	0.406		0.00

## Data Availability

Not applicable.

## Supplementary Materials

The following supporting information can be downloaded at: https://www.mdpi.com/article/10.3390/ijerph192214653/s1.
