# Supplementary material for: Spatial Distribution, Ecological Risk Assessment and Source Analysis of Heavy Metals Pollution in Urban Lake Sediments of Huaihe River Basin
Source: Int J Environ Res Public Health. 2022 Nov 8;19(22):14653. doi: 10.3390/ijerph192214653 (PMC9690674; doi:10.3390/ijerph192214653)
Supplement: Supplementary file 1 [file ijerph-19-14653-s001.zip › ijerph-1987323-supplementary.pdf]

Supplementary Information

1. The in-situ parameters of Cr, Ni, Cu, Pb and Zn concentrations were measured by instrument

The five elements were tested using the XFS method based on DZ/T0279.1,10-2016 and DB34/T 2127.2-2014. The instrument was ZSX PrimusII, the sample quality was 5.00 g, the determination atmosphere was vacuum, the ambient temperature was set at 24 ºC, the ambient humidity was 42%, the carrier gas was argon methane, the voltage was 55 kV, the current was 60 mA, the carrier gas flow was 6.7 mL/min.

2. The in-situ parameters of Cd concentrations were measured by instrument

This element was tested using ICP-MS according to based on DZ/T0279.5.7-2016. The instrument was XSERIES2, the mass of sample was 0.0500 g, the volume was 35 mL, the ambient temperature was set at 23 ºC, the ambient humidity was 50%, the high frequency power was 1.25 kW, the atomization gas flow was 0.80 L/min, the peristaltic pump rotate speed was 30 rpm.

3. The in-situ parameters of As and Hg concentrations were measured by instrument

The sediment samples were digested in water bath with HCl-HNO_3 mixture, then pretreated with thiourea (CH_4N_2S), ascorbic acid ($C_6H_8O_6$) and tartaric acid ($C_4H_6O_6$), and then the total As concentration was determined. Besides, the sediment samples were digested with aqua regia in a water bath, pretreated with potassium dichromate (K_2CrO_4), and then the total Hg concentration was determined. These two elements were tested using AFS based on DZ/T0279.13-2016 and DZ/T0279.17-2016. The instrument was AFS-3100, the sample mass was 0.5000 g/50 mL, the ambient temperature was set at 21 ºC, the ambient humidity was 55% , the negative high pressure was 250 V, the lamp current was 28 mA, the carrier gas flow was 400 mL/min.
